# Incidental Diagnosis of Heterotaxy Syndrome With Left Isomerism in a Postpartum Woman Presenting With Dyspnea and Cyanosis

**DOI:** 10.7759/cureus.117408

**Published:** 2026-10-03

**Authors:** Kunal Sharma, Samen Sharma, Akarshi Gupta, Anjali Prakash

**Affiliations:** 1 Radiodiagnosis, Maulana Azad Medical College, New Delhi, IND

**Keywords:** congenital heart disease, heterotaxy syndrome, left isomerism, polysplenia, postpartum dyspnea, situs ambiguus

## Abstract

Heterotaxy syndrome (situs ambiguus) is a rare congenital disorder characterized by abnormal thoracoabdominal organ positioning. Left isomerism (polysplenia syndrome) is a subtype associated with bilateral left-sided morphology, multiple spleens, and complex cardiovascular anomalies. Most cases present in childhood, but adults may rarely remain undiagnosed until acute illness unmasks the condition. Timely recognition through cross-sectional imaging is critical, as delayed diagnosis in adulthood is associated with high morbidity and mortality, particularly during physiological stress, such as pregnancy and the postpartum period. We present a case of a 25-year-old postpartum woman who presented with shortness of breath, cough, and cyanosis seven days after delivery. Evaluation revealed an oxygen saturation of 70% on room air and a markedly elevated D-dimer level. Contrast-enhanced CT of the chest and abdomen demonstrated left atrial isomerism, double superior vena cava, interrupted infrahepatic inferior vena cava with azygos continuation, polysplenia, a midline liver, a right-sided stomach, dorsal pancreatic agenesis, and bilateral bilobed lungs. Cardiac anomalies included an atrial septal defect, a ventricular septal defect, discordant ventriculoarterial connections, and pulmonary arterial hypertension. This case highlights that heterotaxy with left isomerism may first present in adulthood during physiological stress, such as pregnancy and the postpartum period, and that CT plays a crucial role in defining the full spectrum of anomalies.

## Introduction

Heterotaxy syndrome refers to an abnormal arrangement of thoracic and abdominal organs that does not conform to the usual pattern of situs solitus (normal arrangement) or situs inversus (mirror-image arrangement). It represents a spectrum of disorders of left-right organ patterning and is broadly classified into right isomerism (asplenia syndrome) and left isomerism (polysplenia syndrome), based on characteristic atrial appendage morphology and associated splenic abnormalities [1,2].

Left isomerism is characterized by bilateral left-sided morphological features, including bilateral morphologically left atrial appendages, polysplenia, interruption of the infrahepatic inferior vena cava (IVC) with azygos or hemiazygos continuation, and bilateral bilobed lungs. Associated congenital cardiac abnormalities may include atrial septal defect (ASD), ventricular septal defect (VSD), atrioventricular canal defects, and discordant ventriculoarterial connections. The wide spectrum of anatomical and cardiovascular abnormalities accounts for considerable variation in clinical presentation and severity.

Most patients with significant cardiac malformations are diagnosed in infancy or childhood. However, individuals with milder or physiologically compensated abnormalities may remain clinically stable and survive into adulthood, with the diagnosis occasionally being made during pregnancy, the postpartum period, or an intercurrent illness [3,4]. In the postpartum setting, dyspnea and hypoxemia may initially raise concern for more common acute cardiopulmonary conditions, such as pulmonary embolism, creating a diagnostic challenge when an underlying congenital anatomical abnormality has not previously been recognized. Cross-sectional imaging is therefore important not only for establishing the diagnosis but also for delineating the full spectrum of thoracoabdominal and cardiovascular abnormalities, guiding management, and helping prevent iatrogenic complications during interventions. We present a case of left isomerism heterotaxy diagnosed in a 25-year-old postpartum woman presenting with severe hypoxemia.

## Case presentation

Clinical presentation

A 25-year-old woman presented seven days after an uncomplicated vaginal delivery with progressive shortness of breath, a dry cough lasting two days, and cyanosis. She had no previously known cardiac or anatomical abnormalities. Her antenatal period was reportedly uneventful, and she had no history of childhood cyanotic spells, syncope, or exercise intolerance.

Clinical examination and laboratory findings

Clinical examination revealed markedly reduced oxygen saturation (approximately 70% on room air) and cyanosis of the perioral region and nail beds. Her vital signs showed tachycardia (heart rate, 118/min), tachypnea (respiratory rate, 28/min), and borderline-normal blood pressure (110/70 mmHg). Cardiovascular examination revealed a loud P2 and a systolic murmur at the left sternal border. Respiratory and abdominal examinations were normal.

Laboratory findings revealed a markedly elevated D-dimer level (approximately 20,000 ng/mL; normal: <500 ng/mL). Other laboratory parameters were within normal reference ranges.

Initial imaging

Chest X-ray showed cardiomegaly (cardiothoracic ratio: approximately 0.62). Abdominal ultrasonography showed vascular retained products of conception (RPOC) within the uterine cavity and a spleen in the right hypochondrium. Given the clinical presentation of postpartum dyspnea, cyanosis, and a markedly elevated D-dimer level, pulmonary embolism (PE) was suspected. Contrast-enhanced CT (CECT) of the chest and abdomen was performed using a 128-slice multidetector CT scanner.

Radiological findings

CECT of the chest (Figure 1) showed a predominant left isomerism pattern, characterized by cardiomegaly with a normally positioned heart in the left hemithorax and an apex formed by the right ventricle and pointing to the left, bilateral left atrial morphology (narrow tubular appendages), double superior vena cava, bilateral superior and inferior pulmonary veins connecting to the right-sided atrium, a defect in the fossa ovalis region with a common atrioventricular junction on the left side (ASD), pulmonary arterial hypertension (PAH), dilated coronary arteries, bilateral hyparterial bronchi, bilobed lungs (one fissure on each side), and a bronchial angle of approximately 105° (Figures 2, 3). However, no imaging evidence of pulmonary thromboembolism was identified.

**Figure 1:**
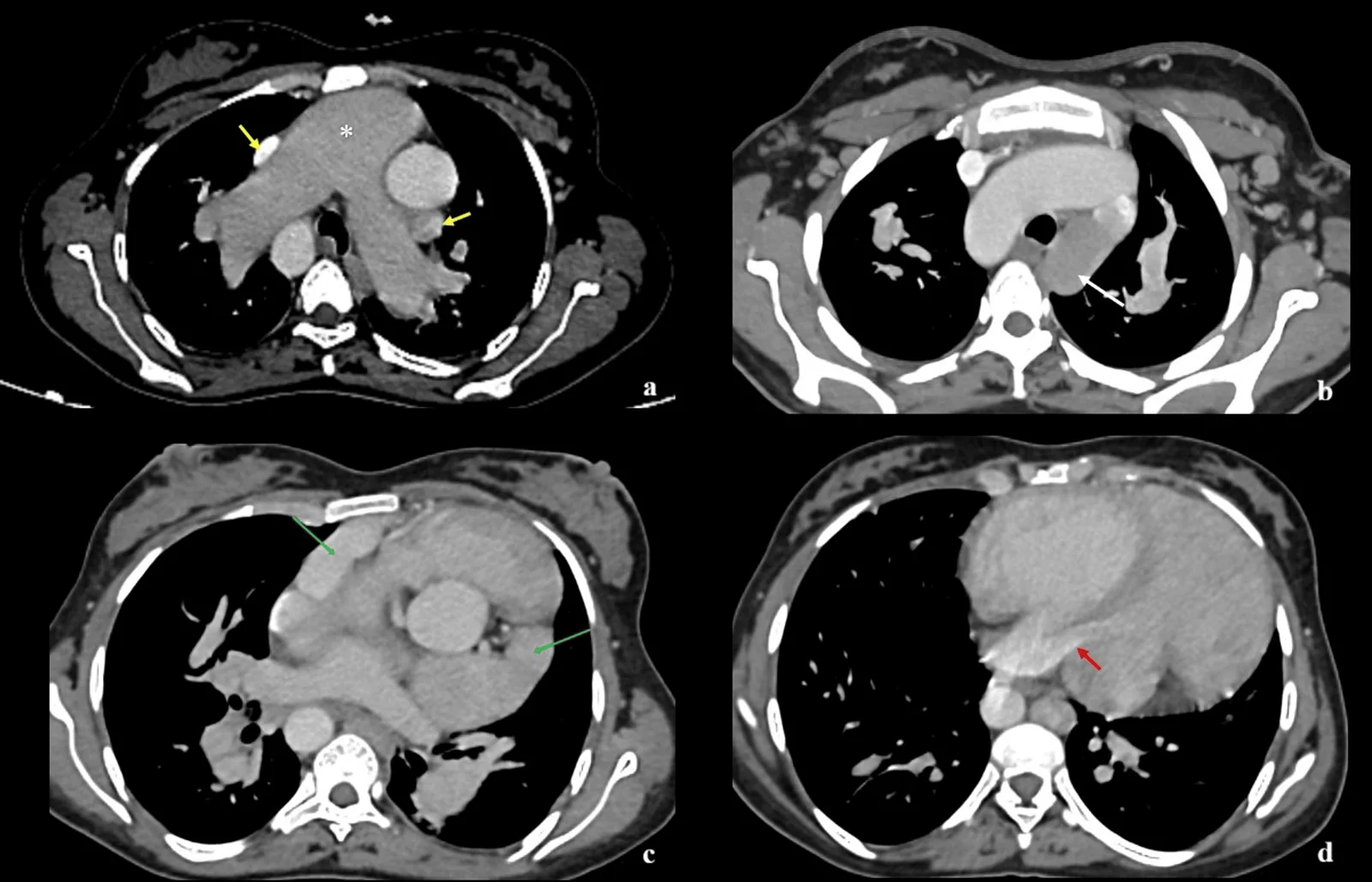
Axial CECT of the chest: (a) double SVC (yellow arrows) and dilated pulmonary arteries (asterisk); (b) left-sided azygos continuation of the IVC draining into the left-sided SVC (white arrow); (c) bilateral narrow tubular appendages suggestive of left atrial isomerism (green arrows); and (d) an interatrial septal defect suggestive of ASD, with a common atrioventricular channel (red arrow). ASD, atrial septal defect; CECT, contrast-enhanced CT; IVC, inferior vena cava; SVC, superior vena cava

**Figure 2:**
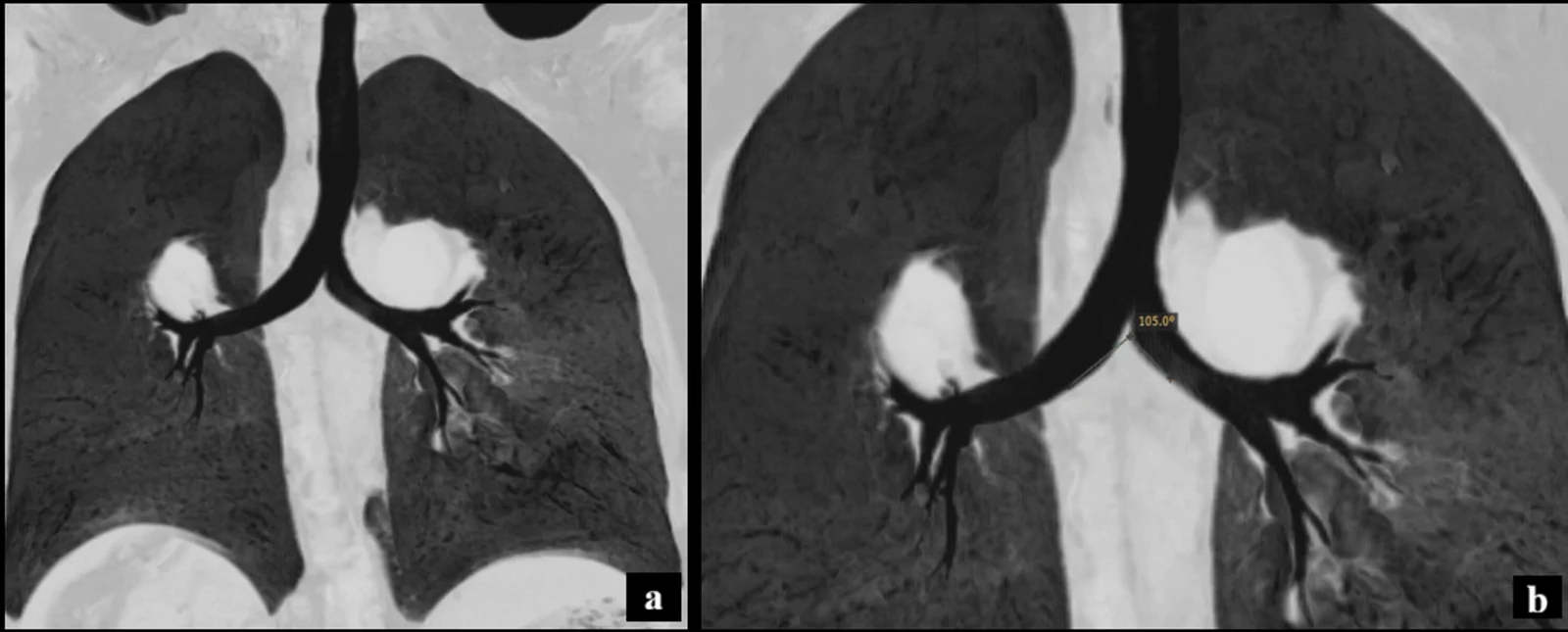
Coronal mini-maximum intensity projection chest reformats showing bilobed hyparterial bronchi and a bilobed bronchial configuration with a carinal angle of approximately 105° (a, b).

**Figure 3:**
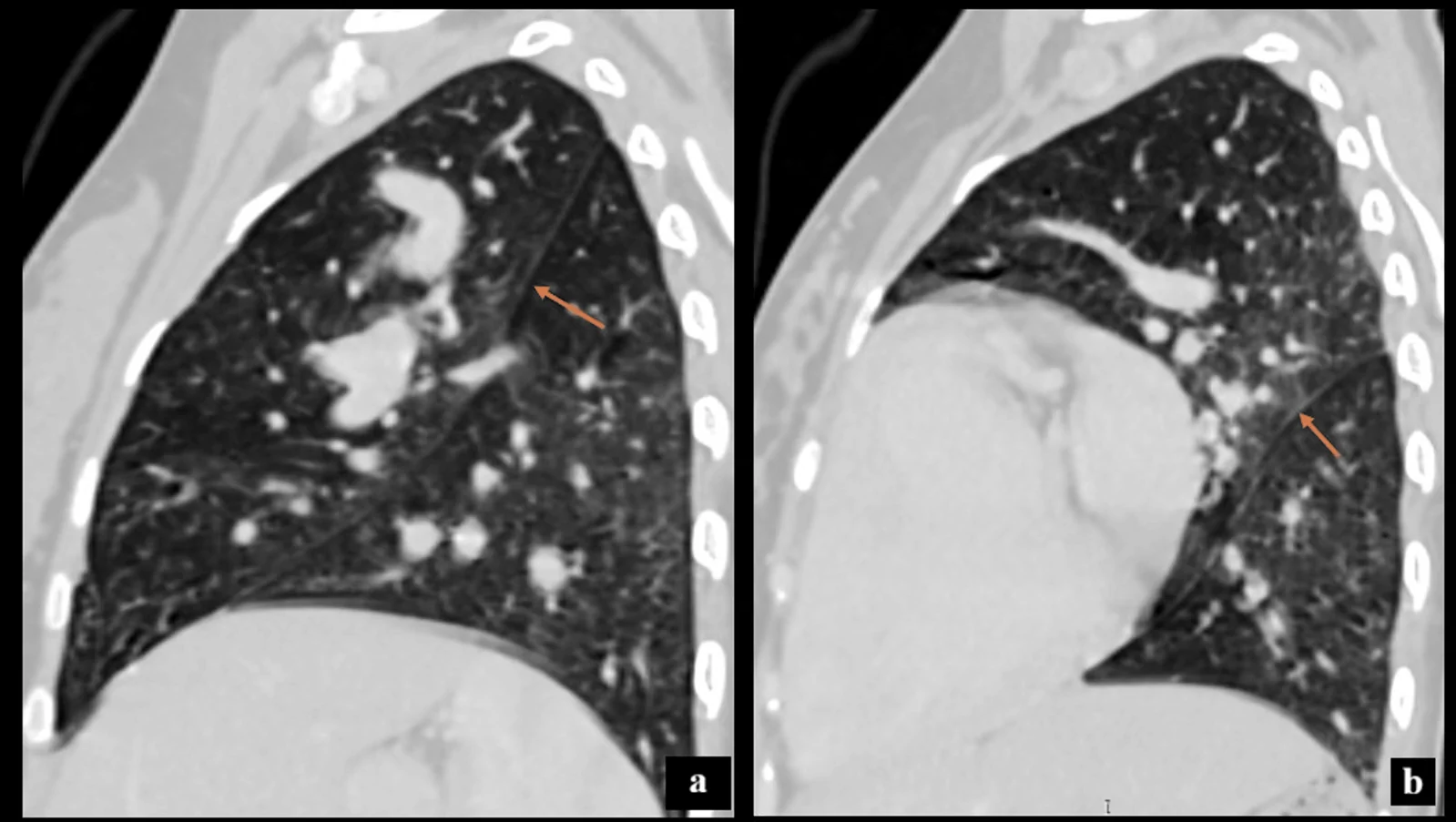
Sagittal CECT images of the chest (lung window, a and b) showing one fissure each in the right and left lungs, respectively, consistent with a bilobed configuration (orange arrows). CECT, contrast-enhanced CT

CECT of the abdomen revealed a midline-positioned liver, a right-sided stomach displaced posteroinferiorly by the liver, polysplenia in the right hypochondrium, dorsal pancreatic agenesis, a left-sided IVC with an interrupted infrahepatic segment and dilated azygos continuation (prevertebral on the left) draining into the left SVC, a dilated portal vein, a posterior nutcracker configuration of the right renal vein, no evidence of intestinal malrotation, vascular RPOC in the uterine cavity, and bilaterally engorged pelvic vessels (Figures 4, 5).

**Figure 4:**
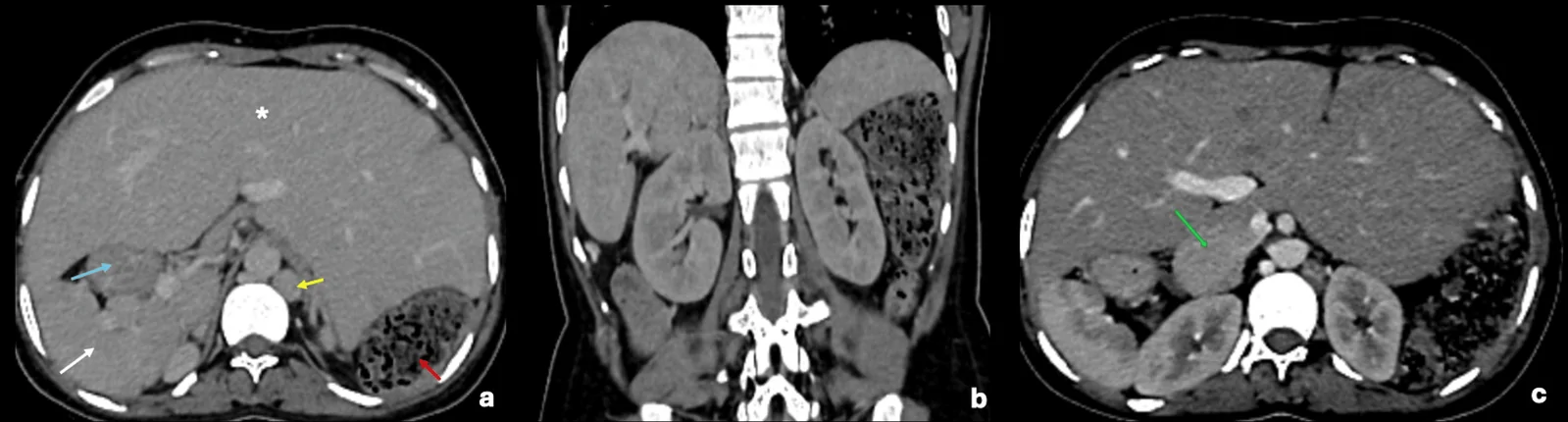
Axial CECT images of the abdomen showing a midline transverse liver (asterisk), multiple splenules on the right side (white arrow), a right-sided stomach (blue arrow), a left-sided IVC (yellow arrow), and the descending colon on the left side (red arrow) (a, b), as well as a normal pancreatic head with a shortened body and tail, suggestive of dorsal pancreatic agenesis (c). The kidneys are normal. CECT, contrast-enhanced CT; IVC, inferior vena cava

**Figure 5:**
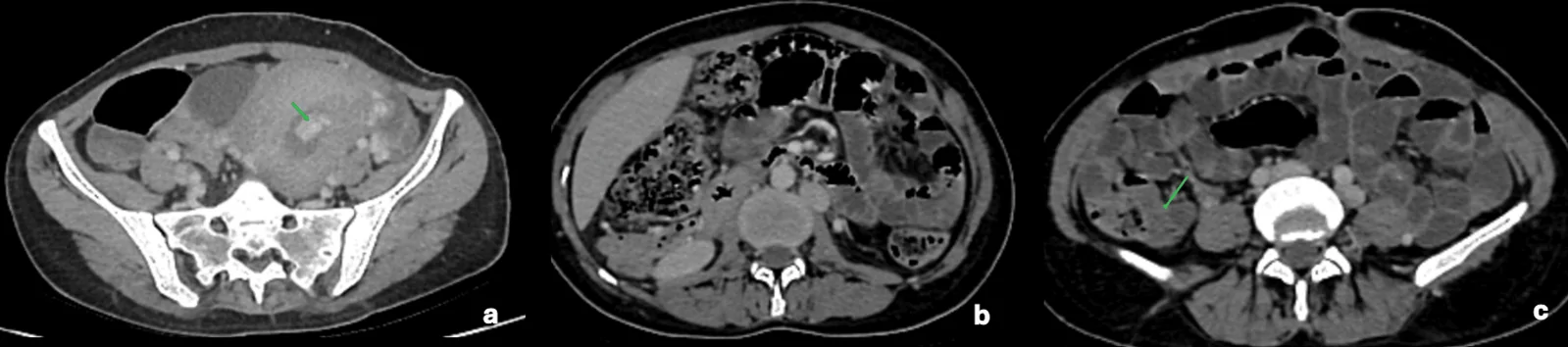
Axial CECT images of the abdomen showing vascular RPOC in the uterine cavity (a), a normal superior mesenteric artery-superior mesenteric vein relationship and a duodenojejunal flexure on the left side (b), and a normally positioned ileocecal junction in the right iliac fossa (c). CECT, contrast-enhanced CT; RPOC, retained products of conception

Two-dimensional echocardiography was performed because of the situs abnormalities and revealed situs inversus, an ASD, a VSD, and PAH. These findings were considered characteristic of heterotaxy syndrome with predominant left isomerism associated with polysplenia.

Subsequent management

The patient was unaware of this anatomical variation and was admitted to the ICU. Cardiology and cardiothoracic surgery consultations were obtained. Echocardiography confirmed severe PAH with a bidirectional shunt. Given her postpartum status and suspected RPOC, a gynecological evaluation was performed. The retained products were managed medically. Anticoagulation was initially withheld because of the risk of hemoptysis from bronchial collaterals. The patient was started on pulmonary vasodilator therapy and supplemental oxygen. Staged cardiac repair was planned following recovery from the postpartum period. Unfortunately, the patient succumbed to her condition in the ICU.

## Discussion

This case represents a rare example of left isomerism heterotaxy syndrome presenting for the first time in a 25-year-old postpartum woman. Most patients with such complex cardiac anomalies (discordant ventriculoarterial connections, ASD, VSD, and PAH) are diagnosed in infancy. Survival to adulthood without diagnosis is exceptional and likely reflects balanced physiology that decompensated under the hemodynamic stress of pregnancy and delivery [5,6]. Only 10% of patients with the polysplenic form of heterotaxy syndrome reach adulthood without complications [5].

Multiple case reports have described heterotaxy with polysplenia, demonstrating considerable variation in the associated anatomical features; therefore, a single definition may not adequately describe the condition. Identifying these variations using imaging is important for preventing potential risks and complications during surgery and invasive procedures. The literature includes case reports describing findings similar to those in our case, including a right-sided stomach and a midline-positioned liver. However, our patient had no intestinal malrotation. In addition, inferior vena cava anomalies are common in patients with heterotaxy syndromes. With the advent and increasing availability of newer imaging modalities, the incidence of diagnosed heterotaxy syndromes may increase, including through screening of otherwise healthy patients. Therefore, recognizing heterotaxy syndromes when normal anatomy is absent, understanding that multiple organ systems may be involved, and making management decisions through a multidisciplinary approach are essential [5,7].

Clinical implications

Role of Early Imaging in Improving Patient Care

Heterotaxy should be suspected in adults with unexplained cyanosis, polycythemia, or atypical situs findings on imaging. Early cross-sectional imaging should be considered even when PE is suspected, as it can simultaneously exclude PE and identify congenital anomalies such as heterotaxy. CT angiography is the modality of choice for defining the full spectrum of cardiovascular and visceral anomalies. Multidisciplinary management involving cardiology, cardiothoracic surgery, radiology, and gynecology is essential. Pregnancy in patients with undiagnosed heterotaxy carries high maternal and fetal risks. Delaying imaging while awaiting echocardiography or serial laboratory tests may worsen outcomes.

Preventing Misdiagnosis

Elevated D-dimer levels and dyspnea in postpartum women often prompt clinicians to initiate anticoagulation for suspected PE. Early CT can help prevent unnecessary anticoagulation in patients with heterotaxy who may have bronchial collaterals or an increased risk of bleeding [6-8].

## Conclusions

Heterotaxy syndrome with left isomerism can remain undiagnosed until adulthood and may first become clinically apparent during the postpartum period. This case emphasizes the importance of considering congenital situs abnormalities in adults presenting with unexplained hypoxemia or cyanosis, especially when the clinical presentation raises suspicion for more common postpartum cardiopulmonary conditions, such as PE. A high index of suspicion and timely cross-sectional imaging in adults with unexplained cyanosis or hypoxemia can establish the underlying anatomical abnormalities, facilitate risk stratification and appropriate clinical assessment, help avoid inappropriate or potentially harmful interventions (such as empiric anticoagulation), and support multidisciplinary management. Despite recognition of the underlying abnormality, patients with complex heterotaxy and associated pulmonary hypertension may remain at substantial risk of adverse outcomes during periods of physiological stress, including the postpartum period. Early recognition and careful, individualized management may therefore be important for optimizing clinical care in such patients.
